# How Children with Autism Spectrum Disorder, Developmental Language Disorder, and Typical Language Learn to Produce Global and Local Semantic Features

**DOI:** 10.3390/brainsci10040231

**Published:** 2020-04-11

**Authors:** Allison Gladfelter, Kacy L. Barron

**Affiliations:** Northern Illinois University, DeKalb, IL 60115, USA; kacy@kacybarron.com

**Keywords:** autism spectrum disorder, developmental language disorder, semantic features, word learning, central coherence

## Abstract

A local processing bias, often considered a cognitive style unique to autism spectrum disorder (ASD), may influence the types of semantic features acquired by children with ASD and could contribute to weaknesses in word learning. Children with developmental language disorder (DLD) also struggle to learn semantic aspects of words, but this cognitive style has not been ascribed to children with DLD. The purpose of this study was to explore whether global–local processing differences influence the type of semantic features children with ASD, DLD, and their neurotypical peers learn to produce when learning new words. Novel word definitions produced by 36 school-aged children (12 with ASD, 12 with DLD, and 12 with typical language) who participated in an extended word-learning paradigm were used to extract newly learned semantic features. These semantic features were then coded for global and local attributes and analyzed to detect whether there were differences between groups. Results indicated that the children with ASD and DLD produced more global, rather than local, semantic features in their definitions than the children with typical language. An over-reliance on global, rather than local, features in children with ASD and DLD may reflect deficits in depth of word knowledge.

## 1. Introduction

Currently, there are ongoing conversations over whether autism spectrum disorder (ASD) and developmental language disorder (DLD) are different ends on a continuum of the same disorder [1,2,3]. Shared traits and similar performance on language tasks perpetuate this discussion. For instance, children with ASD perform poorly on the nonword repetition task [4], a hallmark weakness for children with DLD [5]. Although DLD is primarily characterized by deficits in morphosyntax, tense marking is also impacted in children with ASD [4,6]. Pragmatic deficits are a clinical marker for ASD, but children with DLD can display social communication weaknesses as well [7]. This overlap leads practicing clinicians to report that ASD and DLD can make for a “difficult differential diagnosis” [8]. This challenge is exacerbated when children with DLD also meet the clinical standards for a diagnosis of ASD on the social or communication domains of the Autism Diagnostic Interview, Revised (ADI-R) or the Autism Diagnostic Observation Schedule (ADOS [9]) or both [10].

Efforts to uncover distinct patterns of errors on these language tasks have made some headway in identifying key differences between ASD and DLD. For example, specific patterns of error have been found between groups of children with ASD and DLD on the nonword repetition task [11]. Even though morphosyntactic deficits have been reported in children with ASD, these errors may not include the morphological omission errors that are characteristic of DLD [12]. In a comprehensive review by Williams, Botting, and Boucher [13], further distinctions are described in great detail, such as the widespread phonological deficits in DLD but not in ASD (however, phonological short-term memory deficits have been found in both disorders [14]). These efforts to distinguish between ASD and DLD are essential to elucidate unique language profiles that could aid in earlier and more accurate differential diagnosis.

These challenges in distinguishing between ASD and DLD persist even after the Diagnostic and Statistical Manual of Mental Disorders (DSM-5) revisions were designed to improve accuracy of diagnoses. For a diagnosis of ASD, deficits in social communication and restricted or repetitive behaviors must be present [15]; however, neither of these deficits is necessary for a diagnosis of DLD. Furthermore, ASD must be ruled out to meet the criteria for DLD. As defined by Leonard [16], DLD is a “significant deficit in language ability” for one’s chronological age not caused by hearing loss, nonverbal intelligence, or other neurological deficits (p. 3). Moreover, both groups often perform similarly on tasks outside of the language domain, such as on tasks of motor skill [17]. Because commonalities between ASD and DLD exist, clinicians are often forced to rely on areas known not to overlap, such as restricted or repetitive behaviors, to make a differential diagnosis.

With this high degree of symptom overlap, it is possible that global–local processing differences may be used to help differentiate these two disorders. Individuals with ASD are described as having a cognitive style that lends itself to local processing more than gestalt, or global processing [18,19,20]. This cognitive style is labeled as weak central coherence, or the reduced ability to pull information “together for higher-level meaning” [19,21,22]. This local processing bias is a tendency to focus on small details rather than larger, or more global contexts [19]. In the linguistic domain, this difficulty, i.e., “seeing the forest for the trees”, impacts one’s ability to engage in everyday tasks, such as following along with a story [23,24,25] or applying a shape cue when learning words [26,27,28,29]. Although global–local processing has been widely measured in individuals with ASD, it has been less frequently, or at least more indirectly [30,31,32,33], assessed in children with DLD. When it has been explored, children with DLD have not consistently shown a global or local preference [2,30].

Understanding how children with ASD handle global and local information during tasks of word learning is paramount to developing more effective language interventions. For example, in typical development, toddlers quickly recognize that objects with the same global shape have the same word label [34]. By 24 months of age, these children apply this global shape cue to extend word labels more readily than local cues, such as texture or color [35]. However, this facilitative “shape bias” cue based on global processing has not been found in young children with ASD [26,27,28] or in school-aged children with ASD who have been described as low-functioning [29], showing how the prioritization of local over global processing may contribute to the deficits in word-learning often reported in children with ASD [8,36,37]. Differences in global and local processing also may impact which relevant semantic features of words children with ASD acquire as they form abstract mental representations, or prototypes, of words in their memory. Typically developing infants utilize these abstract prototypes for early categorization [38], and these prototypes are often based on the global shape cue because shape is the most pertinent cue for early object categorization. Perhaps, then, it is unsurprising that children with ASD do not apply abstract prototypes on word categorization tasks [39] or word fluency tasks [40] if they do not attend to pertinent global semantic cues.

Although global shape cues are valuable for early word learning, acquiring the local, detail-specific semantic features of words as children build semantic representations in their mental lexicon is also a fundamental step in developing more complex aspects of language, such as recognizing the salient aspects of words, understanding multi-meaning words, forming sentences, using figurative language and humor, and producing narratives, all areas of difficulty for children with ASD [23,24,25,39,40,41,42]. As children establish semantic representations of words, global semantic features could reflect word referents as a whole, such as describing a cow (basic level) as an animal (superordinate level) or as a heifer (subordinate level). Local semantic features may pertain to a part or detail of the word referent, as in describing a zebra as having stripes. Distinctive semantic features have been shown to aid successful word retrieval in typical learners [43]. However, children with ASD have been reported to acquire fewer semantic features on word-learning tasks than their typical peers [44] and, for those with concomitant syntactic deficits, show sparser word knowledge [8], which may further hinder their ability to successfully produce words. Discovering facilitative ways to teach children with ASD new words seems especially impactful for improving their quality of life, considering that nearly 20% of children with ASD produce fewer than five words on a given day [45]. For clinicians, knowing how children with ASD and DLD acquire global and local semantic features would inform how best to teach new words in intervention, which could have diffuse benefits in their overall language comprehension and use. However, to date, no study has explored how children with ASD and DLD learn to produce global and local aspects of words.

### 1.1. Global–Local Processing in ASD

Performance consistent with the weak central coherence hypothesis has been observed in individuals with ASD on verbal [18,24,25,46,47,48], as well as non-verbal [49,50,51,52,53,54] tasks. In fact, some have suggested that this local bias is a core component of the ASD phenotype [55,56]. Because the weak central coherence hypothesis proposes this cognitive style impacts those with ASD, regardless of age, intelligence, and language ability [19,46,57], global–local processing differences may serve as a potential way to bypass the language commonalities often observed across ASD and DLD to help successfully differentiate between these two disorders.

Local biases influence language productions in ASD. Although this global–local difference has primarily been observed at the level of processing, it is important to determine whether there is any impact on the language productions of individuals with ASD. In a study by Fitch, Fein, and Eigsti [18], adolescents with and without ASD were asked to describe oil paintings by famous artists under a cognitive load (tapping with an index finger). The group with ASD produced as many global details as the other groups; however, the adolescents with ASD still made more local observations than those with typical development, as well as adolescents who had overcome an earlier ASD diagnosis (i.e., optimal outcome; for more information on optimal outcome in children with ASD, see [58,59]). The local bias was apparent in individuals with ASD during this language production task as well.

Booth and Happé [57] utilized a sentence completion task to compare local biases in children and young adults with ASD, typical language development (TLD), and attention deficit hyperactivity disorder (ADHD). On this task, individuals were asked to finish a sentence prompt (e.g., In a cave lived a bat and...), and then their responses were coded as either showing global integration of the over-arching sentence meaning (i.e., a response such as bear or spiders) or local biases (i.e., a response such as ball). Using this language production task, the individuals with ASD were more likely to produce a response with a local bias than their age- and IQ-matched typical peers, as well as their peers with ADHD (to rule out executive function/inhibitory skills as a contributing factor to locally-biased responses). Language production tasks may be used to uncover the local processing bias proposed to reflect weak central coherence in individuals with ASD.

### 1.2. Global–Local Processing in DLD

Although global–local processing in children with ASD has been extensively studied, less is known about global–local processing in children with DLD. To determine if children with DLD have visuo-spatial processing deficits specific to local and global processing, Akshoomoff, Stiles, and Wulfeck [30] compared the performance of children with DLD and typically developing children on the Hierarchical Forms memory task and the Rey–Osterrieth Complex Figure (ROCF) task. The Hierarchical Forms task required the participants to examine visual stimuli constructed in such a way that a larger symbolic image is made up of many smaller symbols that differed from the larger symbol. On this task, the children with DLD were less accurate than the typically developing group overall, but the groups did not differ in accuracy with respect to global and local levels. The authors concluded that the children with DLD, “may adopt simpler or more immature processing strategies… but global or local processing would not be selectively affected” [30].

The results for the ROCF task were similar to the Hierarchical Forms task. The ROCF task required the groups to reproduce a drawing from memory, and performance on this task is known to correlate with visuospatial processing abilities. The children in the DLD group drew fewer details, less accurate figures, and more incorrect cluster placement than the control group on the ROCF task. The authors concluded that the children with DLD relied on a less accurate, immature strategy when copying the figure. Even though these findings exemplify a different pattern of visuo–spatial processing in children with DLD, their performance did not directly reveal differences in global–local processing from their typical peers [30]. If individuals with DLD process global and local information typically (albeit more immaturely), global–local processing tasks may be a viable way to clinically differentiate between ASD and DLD.

### 1.3. Comparing Global–Local Processing in Children with ASD to those with DLD

Global–local processing on linguistic tasks in children with ASD compared to those with DLD has led to mixed findings. In one study, Norbury [31] administered a lexical ambiguity task. In this task, words with ambiguous meanings (e.g., bank) were embedded in sentences given to children with ASD and typical language, ASD and language impairment, DLD, and TLD who had to use context clues to determine which meaning was appropriate (e.g., John stole from the bank). Participants were then shown a picture that was either congruent or incongruent with the meaning best reflected in the sentence and asked to respond “yes” or “no” if the picture matched. According to the weak central coherence hypothesis, individuals with ASD, regardless of language abilities, should show difficulty extracting meaning from broader contexts [19,46]. However, language ability, rather than autism spectrum status, was a better indicator of performance on this task. This well-designed study provides some evidence that the challenges observed in individuals with ASD often attributed to weak central coherence may be better explained by deficits in lexical and semantic knowledge [31].

More recently, Riches and colleagues [32] explored whether autism status or language ability better reflected weak central coherence using a similar forced-choice syntactic ambiguity task with adolescents with ASD and typical language, ASD and language impairment, DLD, and TLD. Unlike the Norbury [31] findings, neither autism status nor language ability led to any significant differences in performance on this linguistic processing task. However, because both studies administered a forced choice task, it is possible that the use of a more open-ended approach would have led to different outcomes.

Although not intended to be a comparison between subgroups of children with ASD with and without language impairments, the open-ended Sentence Completion Task utilized by Booth and Happé [57] included children with autism and children with Asperger syndrome based on the DSM-IV diagnostic criteria, which included a history of spoken language delay for a diagnosis of Autistic Disorder but required an absence of developmental language delay for a diagnosis of Asperger’s Disorder. In this study, both groups of children showed local biases compared to their age- and IQ-matched peers, providing some evidence that autism-status, rather than language or IQ, plays a more influential role in whether or not a child will demonstrate a local-bias on an open-ended, linguistic production task.

In summary, weak central coherence might be a differentiating characteristic between children with ASD and those with DLD. To capture these global–local processing differences, previous studies have primarily employed standardized assessments [30,49,53], magnetic resonance imaging [51,60], switching tasks [54], and scripted sentences or stories followed by a forced choice set of answers [31,32], none of which use the open-ended approach recommended by Happé [22] to best evoke differences in global–local processing. Unlike a labeling, forced choice, or recognition task, open-ended production tasks require the participant to formulate his or her own answers. If global–local processing differences exist between children with ASD and those with DLD, an open-ended task would likely best elicit these differences.

### 1.4. Research Question

In the current study, we embarked on a more open-ended approach. This investigation aimed to explore whether differences in the production of global and local semantic features in a definition task of newly learned, novel words could be used to differentiate children with ASD from those with DLD and TLD. Additionally, knowing how these intrinsic-to-the-learner processing differences impact how children acquire new words is a vital component in better facilitating language learning in these populations. Because children with ASD show a bias toward local details when processing new information, we predicted that they would produce more local semantic features than their peers with DLD and TLD during a novel word definition task; the children with DLD and TLD were expected to produce similar amounts of local and global semantic features.

## 2. Materials and Methods

To explore how children produce global and local semantic features of newly learned words, data collected during previously conducted novel word-learning studies in children with DLD and TLD [61] and with ASD [62] were used for the current study. These original word-learning studies investigated the influence of enriched semantic input on the ability of children with ASD, DLD, and TLD to learn novel words over time. This same data set has also been used to compare how children with ASD, DLD, and TLD acquire visually and verbally presented semantic features during tasks of novel word-learning [63]. In the current study, these novel word definitions were used to determine if the production of global and local features differed by group, potentially shedding light on how local-processing biases influence word-learning in ASD. All of the original recruitment and experimental procedures implemented in the novel word-learning investigations, as well as the analytic procedures and data management for the current study, adhered to the ethical standards approved by each university’s ethical review committee.

### 2.1. Participants

To determine the appropriate sample size for the current study, G*Power statistical software [64,65] was used to conduct a power analysis. For this power analysis, an alpha level of 0.05, power of 0.80, and a moderate effect size of 0.25 were entered as the set parameters for a repeated measures ANOVA with the within (three processing levels) by between (three groups) interaction designated as the planned statistical test. This analysis indicated that a minimum total sample size of 36 would be sufficient. Thus, data from 36 children, 12 children with ASD, 12 children with DLD, and 12 children with TLD, from the original word-learning studies were used for this follow-up study exploring global–local feature productions. All children were recruited from Tippecanoe County, Indiana, USA, and its surrounding counties. For inclusion in the original studies, all participants must have passed an oral-mechanism examination, showed hearing within normal limits on a bilateral pure tone hearing screening, achieved a standard score of 85 or higher on a nonverbal IQ test, and were monolingual English speakers.

Because the previous and current investigators were primarily interested in the production, rather than the comprehension, of newly learned semantic features, and because expressive vocabulary is more reliably measured than receptive vocabulary in children with ASD [66], the expressive vocabulary of each group was compared using raw scores from the Expressive Vocabulary Test-II [67] to ensure the groups did not significantly differ on this key measure (see Table 1). Consistent with previous work indicating that expressive vocabulary is an area of weakness in children with ASD [37] and DLD [68], this matching procedure led to a group with TLD who was significantly younger than the groups with ASD (*p* < 0.01) and DLD (*p* = 0.04). Because the number of locally-biased responses on open-ended production tasks of central coherence has not been shown to differ based on age [57], the data from this original TLD group were still included for comparison. The two clinical groups (ASD and DLD) did not significantly differ in age from each other (*p* = 0.17). Also, because children with ASD show relatively greater impairment in comprehension than production [69], a paired samples *t*-test was conducted to check for differences between expressive and receptive vocabulary in these children. A paired samples *t*-test comparing standardized scores on the Expressive Vocabulary Test—2nd Edition (EVT-2) and the Peabody Picture Vocabulary Test-4 [70] did not reveal any significant differences between receptive (M = 98.42, SD = 18.96) and expressive vocabulary (M = 95.75, SD = 7.57) in the children with ASD, *t*(11) = −0.56, *p* = 0.59. Table 1 depicts a summary of the participant characteristics in all three groups.

The children with ASD were initially recruited for a study exploring the role of semantic richness in word-learning in these children [62]. For inclusion in this original study, the participants with ASD must have a reported independent medical diagnosis of ASD. Then, as part of the inclusionary testing, a trained clinician administered the Autism Diagnostic Observation Schedule—2nd edition [71] to each participant with ASD to confirm that they met the cut off scores for either autism or the autism spectrum. All of the children with ASD included in the original studies were verbal communicators who did not use any form of augmentative or alternative communication as a primary means of communication. Following these inclusionary testing procedures, 12 children (three females) with a mean age of 7; 9 (years; months, range 4; 6–11; 3) were included with ASD. One participant (ASD1) was unable to complete the nonverbal IQ test due to a behavioral rigidity that led to the consistent selection of items in the same location from the array of choices. Because ASD1 was able to successfully participate in the experimental word-learning tasks, her expressive vocabulary score was similar to participants with DLD and TLD, weak central coherence is not hypothesized to depend on intelligence [19], and intelligence has not been shown to be a significant factor on open-ended tasks exploring central coherence [57], her data were still included in the current study. After meeting all inclusionary criteria, the Structured Photographic Expressive Language Test—Third Edition [72] or the core battery of the Clinical Evaluation of Language Fundamentals—4th Edition [73], whichever was age appropriate, was administered to eleven of the children with ASD to capture their broader expressive language skills. Due to time constraints, one participant with ASD was not given either expressive language test.

The children with DLD and TLD were originally recruited to participate in a multi-year, longitudinal study exploring the relationships between motor and language skills [61,74,75,76,77]. As such, the inclusionary testing procedures for these children were implemented one or two years before the collection of their novel word definitions that were used for comparison in the current study. Inclusion criteria outlined by Leonard [16] were used when qualifying participants for the group with DLD. Specifically, these participants obtained scores at or above 85 on a standardized nonverbal IQ test, demonstrated hearing and oral-mechanism functioning within normal limits, and had no history of a neurological disorder. Additionally, during their initial year in the longitudinal motor and language investigation, each participant achieved a standard score at or below 87 on the Structured Photographic Expressive Language Test–Preschool—2nd edition [78], which has good sensitivity and specificity when diagnosing DLD [79] using the criteria outlined by Greenslade, Plante, and Vance [80]. Finally, to rule out ASD, all children with DLD were assessed with the Childhood Autism Rating Scale—2nd Edition [81] and secured scores within the “Minimal-to-No symptoms” range. Based on these inclusionary criteria, 12 children (three females) with a mean age of 7; 1 (range 5; 9–8; 4) were included with DLD in the current study.

To be included in the group with TLD in the original longitudinal study, parental reporting was used to confirm that the children had no history of language delays. Also, the children had to have achieved a standard score of 85 or higher on either the Structured Photographic Expressive Language Test–3rd edition [72] or the core battery of the Clinical Evaluation of Language Fundamentals–4th edition [73], depending on which was age appropriate at the time of their initial inclusion in the longitudinal study. Finally, all children with TLD received scores within the “Minimal-to-No symptoms” range on the Childhood Autism Rating Scale—2nd edition [81]. Based on these inclusionary procedures, 12 children (six females) with a mean age of 5; 10 (range 4; 3–7; 3) with TLD were included in the current study.

### 2.2. Auditory Stimuli

Six novel words (/fʌ∫pəm/, /pʌvgəb/, /bʌpkəv/, /mʌfpəm/, /fʌspəb/, and /pʌbtəm/) were presented auditorily to the children in the original word-learning studies [61,62]. These two-syllable phonetic strings were controlled for phonotactic probability and neighborhood density, factors known to affect a word’s learnability [82,83]. All words were recorded by a female native-English speaker and loaded into Praat [84] to equate for intensity at 70 dB Hearing Level. The novel words were presented through a set of external speakers located in front of the participants. Depending on the original semantic cue condition (no semantic cues, sparse semantic cues, or rich semantic cues [62]), recordings of four of these novel words were presented in synchrony with a matched visual referent (i.e., paired word form with meaning) either in isolation (sparse semantic cues condition) or embedded in a children’s story (rich semantic cues condition). Two novel words were never paired with visual-referents (no semantic cues condition) to compare how children produce words given semantic cues to those taught without any semantic information. Only the novel words taught with visual referents (i.e., sparse and rich semantic cues conditions) were included in the current study. All three pairs of novel words were randomized and counterbalanced across participants and groups.

### 2.3. Visual Stimuli

**Figure 1:**
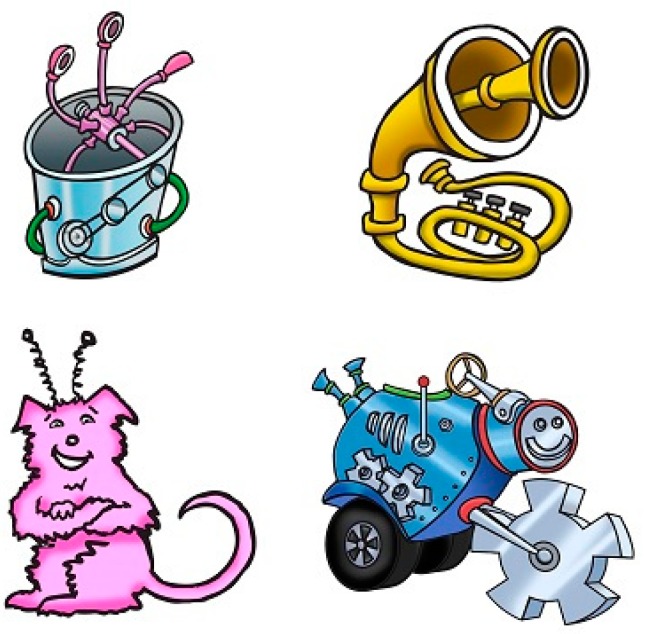
Visual referents used in the original word-learning paradigm [62].

In the original word-learning studies, four child-friendly drawings by a professional illustrator (Figure 1) were used as the visual referents for the novel words [61,62]. Each visual referent came from a distinct superordinate semantic category; a tool, an instrument, an animal, and a vehicle. In the original studies, the tool and instrument referents were taught in the sparse semantic cue condition. In this sparse semantic cue condition, the children were auditorily presented a novel word in synchrony with the visual referent. For the animal and vehicle referents, the novel words were embedded in a children’s story in the rich semantic cue condition. Prior to teaching these visual referents in the semantically enriched condition in the original word-learning studies [61,62], all four visual stimuli were tested in the semantically sparse condition to assess whether any image was inherently more learnable. Based on this testing, no referent was significantly more learnable in any of the original word-learning measures (e.g., referent identification, confrontation naming, phonetic accuracy, or kinematic stability). All visual images were displayed on a 76.2 cm Dell monitor screen placed in front of the children that was connected to a laptop with Microsoft PowerPoint. The children’s story script with all of the corresponding visual images is available in Gladfelter and Goffman [62] and is provided in the Supplementary Materials for this article.

### 2.4. Collection of Word Definitions

The definitions used to extract local and global semantic features were collected following their presentation in either the sparse semantic cues condition (i.e., picture–word pair in isolation) or the rich semantic cues condition (i.e., embedded in a children’s story) in the original word learning studies [61,62]. To control for any primacy or recency effects, the presentation order for the three semantic learning conditions (no semantic cues, sparse semantic cues, rich semantic cues) was counterbalanced across children. These original studies focused on whether the semantic richness of the learning context influenced a child’s ability to acquire new words, whereas the current study expands upon this earlier work by exploring the differences in the types of semantic features the children produced, specifically at the global or local processing level.

In these prior studies, participants were presented novel words seven times on three separate days approximately one week apart (or 21 total exposures per novel word across all sessions). After being presented with the meanings of the novel words in each semantic cue condition, participants were asked to define the novel words using the open-ended examiner prompt, “What does ____ mean?”. After their initial response, all participants received one follow-up prompt, “What else can you tell me about _____?” [85]. These open-ended prompts are unlike some past studies targeting global–local processing (e.g., [31]), which limited their participants to two choices (e.g., “yes” or “no”). Although the original word learning studies were not explicitly designed to target central coherence, open-ended tasks are recommended for assessing the impact of global–local processing in children with ASD [22], making the use of these novel word definitions an ideal method for comparing global and local productions in children with ASD, DLD, and TLD. All definitions were recorded and transcribed for later coding. A total of 432 definitions (36 participants × 4 definitions × 3 sessions) from these word-learning studies provided the data for the current study.

### 2.5. Extraction of the Semantic Features from the Definitions

In the original word-learning studies, the semantic features were extracted from the definitions to score the number of accurate units of information (i.e., the number of semantic features) drawing from the method described in McGregor, Sheng, and Ball [85]. As an example, one child defined the vehicle as follows: “In the story, Big Brother said his /pʌbtəm/ makes donuts ^1^. He said it’s shiny ^2^, and it looks like a motorcycle ^3^ and it goes faster ^4^ and faster!”. This definition contained four accurate units of information about the meaning of the target word. In the original investigation, a second coder was trained to calculate the reliability of the number of accurate units of information produced. For reliability training, the definitions from three randomly selected participants (one from each diagnostic group) were scored separately by both coders for the number of accurate semantic features. Then, within the context of training, disagreements were thoroughly discussed, and consensus building took place. For the reliability scoring, a new set of definitions distributed equally across groups from 25% of all sessions was selected using the same random number generator (random.org) to select the participant numbers. The total number of semantic features identified by the original primary author (Gladfelter) was 270 and by the second coder was 284, with an overlap of 269 semantic features. Reliability was then judged to be between 94.7% (269/284) and 99.6% (269/270). For the current study, the semantic features from all 432 definitions were analyzed based on whether the semantic information was a global or local attribute.

### 2.6. Global and Local Coding of Semantic Features in the Current Study

The semantic features extracted in the original word-learning studies were used in the current study. To prevent bias during coding, the second author was blinded to the diagnostic category of each participant using a de-identifying alphanumeric coding system devised by the first author. A coding manual was designed to promote consistency across coders and to explain the coding process to an undergraduate research assistant for later reliability coding. The second author used a Microsoft Excel worksheet to code the participants’ definitions following manualized rules developed by the authors.

The semantic features were analyzed to see if they reflected a local detail or the global object. Although previous word-learning and categorization studies have used the global shape cue to explore how children apply this category relevant cue to category irrelevant cues (e.g., size, color, or texture) when learning new words, the purpose of the current study was to focus on which semantic features produced by children required processing of the novel referent as a whole or only required the processing of local details, or smaller parts, of the novel referent as they formed semantic representations of the newly-learned words. This use of semantic features produced during a novel word-learning definition task is a new approach to investigating global–local processing. The weak central coherence hypothesis [19,21] proposes that children with ASD show a processing bias for local details at the expense of holistic meaning. This hypothesis has classically been assessed using the Navon Hierarchical Figures Task [86], which presents alphabetic letters composed of smaller alphabetic letters and then determines whether the individual preferentially processes the local parts (smaller letters) or the global whole (bigger letters) of a visual image. Using hierarchical figure tasks, individuals with ASD have been shown to demonstrate a preference for local, rather than global processing, the opposite pattern of those with more typical development [87,88]. To more closely align with this classic global–local task, rather than a word categorization task, we chose to code semantic features that either captured the novel word-referent as a global whole object or as a local part.

To analyze the processing level, the coders determined if each semantic feature was (1) Global (whole object), (2) Local (details or parts), or (3) N/A, indicating coding was not applicable at the global or local level. If the participant provided a semantic feature that described the target referent as a whole, the coders scored it as Global (whole object), or, if the participant produced a semantic feature that described a part or detail of the target referent, the coders scored it under the Local (details or parts) category. For example, if the child said “antennas” for the animal target referent, it was coded under Local because this pertained to a specific attribute of the animal and not the whole. If the child produced a semantic feature such as “pet,” it was marked as Global because it referred to the whole referent. It is worth noting that the global–local coding implemented in the current study was conducted on each of the originally extracted semantic features individually and not on all features provided within a definition collectively. In other words, if a child’s definition provided several detail-specific features that, together, would provide a more holistic description of the referent, these individual features were still coded as Local.

Not every semantic feature was marked for local or global processing because not all semantic features were able to be coded as a global or local attribute (e.g., the semantic feature was an action, emotion, or descriptive word). In this case, the coder scored the semantic feature as N/A for not applicable. For example, the coder marked “N/A” if the child said “gives kisses” to define the animal referent because it could not be separated into global or local parts.

### 2.7. Reliability and Training

To assess the inter-rater reliability of the global/local semantic feature coding, one undergraduate research assistant majoring in Communicative Disorders coded 25% of the definitions (i.e., data from nine participants). These were chosen using a random number generator (www.random.org) to select the participant numbers, with an equal distribution across the three diagnostic groups. The selection of 25% of the total data collected fits within the criteria outlined by Schlosser [89], which recommends inter-rater reliability be conducted between 20%–30% of the total data. The randomly selected set of participants used for the final reliability coding did not include any data used during reliability training and was also de-identified using the same alphanumeric system to blind the undergraduate coder and the second author of each participants’ diagnostic category. To determine inter-rater reliability, Cohen’s kappa was derived before consensus building occurred. Following the ratings described by Hallgren [90], the kappa statistic for the processing-level coding was almost perfect agreement (k = 0.932 with a 95% confidence interval of 0.881–0.983). Disagreements were discussed, and then consensus building took place.

### 2.8. Statistical Analyses

A mixed-model ANOVA was conducted with diagnostic group (ASD, DLD, and TLD) as the between-subjects variable, and processing level (global vs. local vs. not applicable) served as the within-subjects variables. From the original 432 definitions, a total of 817 semantic features, with 257 from the children with ASD, 335 from the children with DLD, and 225 from the children with TLD, were coded. The sum of semantic features within each global, local or N/A coding category was calculated individually for each participant and collapsed across sessions. For the mixed-model ANOVA, these summed totals of responses served as the within-subjects data. An alpha level of less than 0.05 was considered significant.

## 3. Results

This study aimed to determine whether the global or local semantic features produced during a definition task could be used to differentiate children with ASD from those with DLD and TLD. A summary of the results for diagnostic group and processing level effects is presented in Table 2.

### 3.1. Global–Local Processing Level Effects

The mixed-model ANOVA revealed a significant effect based on the global–local processing level (*p* < 0.001). Follow-up least significant difference (LSD) pairwise comparisons indicated that more global than local (*p* < 0.001) semantic features were produced during the novel word definitions. Also, more features were categorized as N/A than as global (*p* < 0.001) or local (*p* < 0.001). Because the primary goal of this study was to assess the influence of global and local processing on the production of semantic features, this significant finding is not further discussed here.

### 3.2. Group and Global–Local Processing Interaction Effects

Although the mixed-model ANOVA did not reveal a significant group effect (*p* = 0.295), it did reveal a significant interaction between diagnostic group and processing level (*p* = 0.030). Follow-up pairwise comparisons (LSD) indicated that the children with DLD produced significantly more global semantic features than their peers with TLD (*p* = 0.012), and the children with ASD approached significance (*p* = 0.054) towards producing more global semantic features than their peers with TLD. The groups with DLD and ASD did not differ from each other (*p* = 0.522) in their production of global semantic features. There were no other significant interactions between groups and local semantic features or features coded as N/A (all *p* values >0.05).

Within each group, the children with ASD (*p* = 0.008) and DLD (*p* < 0.001) produced significantly more global features than local features within their novel word definitions. The children with TLD did not differ in their production of global and local semantic features (*p* = 0.877). All groups of children produced more N/A features than global and local semantic features (all *p* values < 0.05), except for the children with ASD who did not differ in their production of global and N/A features (*p* = 0.224). Because the study aimed to focus on global and local semantic features, these significant N/A findings are not further discussed here. All group means and standard deviations for each processing level are summarized in Table 3, and each participant’s mean number of features is presented in Table 4.

### 3.3. Post-hoc Results Based on Age and Expressive Vocabulary

Because the use of global, over local, descriptive terms during definition tasks has been shown to increase developmentally [91], and the ASD and DLD groups were significantly older than the group with TLD, a follow-up ANCOVA was conducted with age as a covariate. In this post-hoc analysis, there was no significant interaction between level of processing and age, *F*(2, 31) = 1.08, *p* = 0.352.

Furthermore, because some previous studies have reported that language, rather than autism status, is a better predictor of performance on tasks assessing weak central coherence [31], an additional follow-up ANCOVA was conducted with EVT-2 standard scores as a covariate. As with the age results, this post-hoc analysis revealed no significant interaction between level of processing and language performance on an expressive vocabulary test, *F*(2, 31) = 0.60, *p* = 0.553.

## 4. Discussion

Global–local processing differences influenced the type of semantic features produced by children with ASD and with DLD compared to their typical peers on a word learning task, but not in the ways expected. It was predicted that the group with ASD would provide more local features than the group with TLD, and the group with DLD would be similar to the group with TLD in its use of global and local features. However, the groups with DLD and ASD (albeit only approaching significance) both produced more global features than the TLD group. Although these findings were unexpected within the framework of the weak central coherence hypothesis, these outcomes are consistent with a growing body of semantic learning literature [8,44,68,92,93,94,95,96] in children with ASD and DLD, indicating that these children show difficulty acquiring more detail-specific information. These results also align with robust literature on the whole object assumption in early word-learning [97,98,99] in which children assume that object labels refer to an object as a whole rather than individual parts. Furthermore, the results are consistent with some [32], but not all [31], previous work focusing exclusively on weak central coherence in the linguistic domain.

Before interpreting these results more fully, four methodological limitations must be considered. First, because the data were extracted from already completed novel word-learning studies, and because the initial power analysis indicated that the sample size was sufficient, additional participants were not recruited for this study. Although the sample size was large enough to reject the null hypothesis, additional studies beyond this initial exploratory study are needed to replicate and more thoroughly investigate global and local processing’s influence on language production tasks in children with ASD and DLD. Second, because of the original decision to match groups on expressive vocabulary, the groups with ASD and DLD were significantly older than the group with TLD. Although previous work investigating central coherence in individuals with ASD did not find any effects based on age [57] and our post-hoc analysis did not uncover any age-related effects, future research should include a chronological age-matched group with typical language to more directly determine whether developmental maturity is a contributing factor. Third, nearly half of the children with ASD also showed signs of a concomitant language disorder based on standardized language assessments. Perhaps, then, it is unsurprising that no differences were found between the children with ASD and DLD on this language production task. In one previous study, language ability, rather than autism status, was found to impact performance in comprehension tasks comparing global–local processing in children with ASD and DLD [31], suggesting that this may be a contributing factor in this production task as well. However, this finding has not been consistently replicated in later studies employing similar language comprehension tasks of global–local processing [32]. In the current exploratory study, a post-hoc analysis did not reveal any language-related effects based on expressive vocabulary scores, but clearly additional research is needed to fully assess the relationship between receptive and expressive language abilities and global–local processing in children with ASD and DLD beyond this study. Finally, because the current study analyzed already collected data, no measures of non-verbal global–local processing were implemented during the original word-learning studies for comparison to the verbal measures explored in this study. Future research that directly assesses both verbal and nonverbal global–local processing in children with DLD and ASD is necessary to fully determine the influence of verbal semantic weaknesses on tasks of weak central coherence.

### 4.1. The Local Biases in ASD Revisited

We anticipated that the children with ASD would produce an over-abundance of local descriptor words because of their local perceptual biases; however, they unexpectedly produced a similar amount of local semantic features and a trend toward more global features than their typical peers. In hindsight, this should not have been surprising. Traditionally, evidence in support of the weak central coherence hypothesis has focused on visuo-spatial tasks [49,50,51,54], whereas evidence in the linguistic domain has been varied [31,32]. Previous researchers have shown that verbal children with ASD can establish semantic categories for words at the basic and superordinate levels as well as their typical peers [96], recognize typical members of familiar word–object categories [92], and can extend word label categories broadly [95], all tasks that would require them to process word referents at the global level. It is worth noting that, although children with ASD can overcome local biases to acquire globally descriptive terms when learning new words, not all children with ASD do [26,27,28,29].

One reason for this discrepancy in findings could be due to the conceptualization of central coherence. As discussed by Riches and colleagues [32], there are two differing emphases within this hypothesis; either a reduced ability to integrate information or an enhanced ability to focus on local information (p. 156). Linguistic studies more often focus on the integration side of this hypothesis, such as employing tasks that, at the local level, may be ambiguous, but when the information is integrated across the global and local levels, there is a correct interpretation and response. For example, previous work used homographs [46,47], multi-meaning words [31], or sentence fragments [57] that required the listener to pull together contextual information to select the more appropriate pronunciation, word meaning, or phrase. In contrast, studies outside of the linguistic domain focused more heavily on the enhanced processing of local details, such as through the use of the embedded figures task [49] or motion perception tasks [51]. In the current study, the children’s story provided both verbal (linguistic) and visual information, allowing the children to freely rely on whichever learning strategy they naturally would to acquire the semantic features of new words. Interestingly, in a previous study using this same data set [63], these same children with ASD and DLD produced more semantic features that were originally taught in the visual images rather than through the verbal modality alone or in the visual and verbal modalities in combination. Even though both clinical groups of children relied heavily on the visual modality, which would align more closely with the enhanced local processing observed on visual-perception tasks in children with ASD, these same children instead produced more global than local semantic features, which does not provide support for the weak central coherence hypothesis.

Additional methodological differences between the current study and previous weak central coherence investigations may further explain the difference in outcomes. First, the use of child-friendly cartoons, rather than the more visually complex oil paintings used by Fitch and colleagues [18], may have facilitated global–local processing in the children with ASD. Also, the painting descriptions were collected under an increased cognitive load (finger tapping). These differences could explain how the children with ASD in the current study were able to describe the novel words in terms that demonstrated an ability to integrate the local details of the target referent into a whole.

Another key difference could be within the degree of autism symptom severity. Fitch and colleagues [18] found that the current symptoms of their participants with ASD did not relate to global and local focus, but the relative severity of autism symptoms over the lifespan did. Others have found similar symptom severity associations with weaker central coherence on non-linguistic tasks in individuals with ASD [50]. Also, in minimally verbal children with ASD, a lack of a shape bias could also reflect support for weaker central coherence in children with more severe autism-related symptoms. Perhaps the children in the current study, who were all verbal and had nonverbal IQs in the typical range, did not display as severe symptoms and therefore did not present a local bias.

Also, exposure time is a likely factor. Others have posited that individuals with ASD show global perceptual deficits due to differences in visual processing speed and require longer amounts of time to recognize objects as a whole. With additional time to analyze images, individuals with ASD accurately integrate local signals into a global whole [50,51]. In the current study, the word-referent pairs were presented 21 times over the course of three different days roughly a week apart—possibly providing ample time for the children with ASD to process the referents at the global level.

However, an ability to overcome local biases fails to capture why the children with ASD produced more global than local features. Previously, McGregor and Bean [95] sought to determine whether local perceptual biases would lead children with ASD to extend object labels too narrowly during word extension tasks. Instead, the children with ASD who also had concomitant semantic and syntactic language difficulties had established broader word categories when a narrower, more specific category boundary would have been more appropriate. Because nearly half of the children with ASD in the current study showed signs of language weaknesses, perhaps they too acquired more broad labels for the novel words. As Norbury posited in 2005, language ability, rather than autism status, may be a better indicator of one’s ability to synthesize semantically relevant, higher order information.

### 4.2. An Abundance of Global Features in Children with DLD Likely Reflects Semantic Deficits

Surprisingly, the children with DLD produced significantly more global semantic features than the group with TLD in their novel word definitions. These global features only captured the novel objects at the most basic level of detail. As an example, one participant with DLD provided the following definition for the “tool” referent (with coded features in italics): “Bucket ^1^ (global). Blue ^2^ (not applicable), shiny ^3^ (not applicable). Blue. It’s a tool ^4^ (global)”. In comparison, a participant with TLD responded: “Pubtum means like it looks like a bucket ^1^ (global) and it has gears ^2^ (local) in it, and like all these wires ^3^ (local) and it had a spinny thing in the middle ^4^ (local).” Both participants provided four semantic features, but the participant with TLD provided features with a more specific level of detail, giving the semantic representation more depth, whereas the participant with DLD only gave semantic features that described the referent at a more global level.

This reliance on global terms (indicative of knowledge of breadth) over local details (indicative of knowledge of depth) in children with DLD may be that they are compensating for their sparse, less in-depth, semantic representations [68]. This interpretation was illustrated in McGregor and Appel’s [94] study, in which a child with DLD produced fewer detailed, local features and instead substituted for a semantically related word at the same, whole-object hierarchical level (e.g., describing a helmet as a “hat”). Even when defining commonly used nouns, children with DLD define these concepts without much depth [93]. McGregor and her colleagues proposed that these shallower semantic representations in children with language impairments could be because they possess fewer words in their vocabularies compared to their typical peers [68,95]. With fewer words in their mental lexicons, the number of mappings between newly acquired words and words already established would be limited. If children with DLD possess fewer local, detail-level terms within their lexicon, they will continue to be limited in their ability to acquire and integrate the local features of newly learned words. These weaker, less robust semantic representations may also explain why children with DLD show difficulties extracting relevant information from broader linguistic contexts [31].

It is possible that children with DLD do not acquire these more detailed, in-depth semantic representations because of a global, rather than local, processing bias. However, children with DLD have not been shown to prioritize processing global over local information in levels of processing tasks previously [30]. Furthermore, children with DLD, much like those with ASD, show a weaker shape bias during novel object naming tasks than their typical peers [33], making this explanation of a preference for global, over local, processing unlikely.

### 4.3. The Use of Global Features during Word Definition Tasks Changes Developmentally

Alternatively, the children with ASD and DLD, due to their older ages, may be providing a more developmentally advanced definitional form than their younger peers with TLD. The use of global terms demonstrates an ability to consolidate multiple semantic features representing the target referent and therefore is arguably a more mature form to use during a definition task. In contrast, using multiple local details to describe one referent is more immature developmentally [91]. Skwarchuk and Anglin [91] state that superordinates indicate a mature definitional form that improves as children grow older. Because of the methodological decision for matching based on expressive vocabulary, the children with ASD and DLD in the current study were significantly older than the group with TLD, which may be why they included more global descriptor terms; it was developmentally more appropriate. Furthermore, Skwarchuk and Anglin [91] found that nouns elicited more superordinate terms in the children’s definitions than verbs or adjectives. The target referents in the current study were all nouns, which also supports the use of superordinate terms. Rather than reflect a linguistic weakness, the use of global features to describe a noun on a definition task may have been more developmentally appropriate for the older children with ASD and DLD. However, the follow-up ANCOVA exploring a potential interaction between age and processing level of the coded semantic features in the current study was not found to be significant, which makes this developmental explanation for the over-use of global terms in the children with ASD and DLD less likely. However, to more directly address this possibility, future research should include a chronological-age matched sample of participants with typical language.

### 4.4. Clinical Implications

This study contributes to a growing body of literature exploring the qualitative differences in the vocabulary knowledge of children with language impairments. Consistent with the findings of a massive undertaking by McGregor, Oleson, Bahnsen, and Duff [68] analyzing 25,681 definitions produced by school-aged children, the current results found that the children in both of our clinical groups (ASD and DLD) showed signs of limited depth of vocabulary knowledge, as shown by an overuse of global, rather than detailed terms, when defining new words. Further, based on the findings of the study by McGregor and colleagues [68], the older ages of the participants in our study, and work including young adults with specific learning disabilities [100], these semantic deficits persist over time. Even though clinicians often focus on pragmatic language skills in children with ASD and morphosyntactic skills in children with DLD, semantic deficits must also be addressed.

## 5. Conclusions

This study explored whether local processing biases in a word definition task in children with ASD could differentiate them from children with DLD and TLD. When acquiring local and global information, the children with ASD and DLD produced more global semantic features in their definitions compared to children with TLD. This finding does not support the idea that a local processing bias prevents children with ASD from successfully acquiring global semantic information as they learn new words. Because the children with DLD were not expected to show differences from their typical peers in global–local processing, it is unclear whether these global semantic feature production differences are due to global–local processing challenges or simply reflect weaker semantic (depth of word knowledge) skills. Future work is needed to investigate the relative contributions of global–local processing and semantic language skills in the formation of semantic representations during tasks of word-learning in children with ASD and DLD.

## Figures and Tables

**Table 1:** Summary of the participant characteristics.

	ASD (*n* = 12) *M* (Range)	DLD (*n* = 12) *M* (Range)	TLD (*n* = 12) *M* (Range)	*F* Value	*p* Value
Age (years; months)	7; 9 (4; 6–11; 3)	7; 1 (5; 9–8; 4)	5; 10 (4; 3–7; 3)	6.39	0.01
Sex	3 F, 9 M	3 F, 9 M	6 F, 6 M	1.10	0.34
EVT-2 Raw Score	88.67 (53–120)	82.00 (67–97)	94.5 (68–128)	1.41	0.26
EVT-2 Standard Score	95.75 (79–112)	94.17 (78–106)	114.83 (91–135)	15.66	<0.01
Nonverbal IQ Standard Score	96.6 (85–106) *	104.08 (91–125)	121.50 (96–149)	12.88	<0.01
Language Standard Score	86.18 (58–111) *	73.67 (42–87)	112.09 (90–125)	21.63	<0.01

EVT-2 = Expressive Vocabulary Test—2nd Edition; F = female, M = male; ASD = autism spectrum disorder; DLD = developmental language disorder; TLD = typical language development; Nonverbal IQ Standard Scores were from the Primary Test of Nonverbal Intelligence, the Columbia Mental Maturity Scale, or the Test of Nonverbal Intelligence; Language Standard Scores were from the Structured Photographic Expressive Language Test- Preschool–2nd edition, Structured Photographic Expressive Language Test–3rd edition, or the Clinical Evaluation of Language Fundamentals–4th edition; *, only includes scores from 11 participants with ASD. One-way ANOVA with equal variance assumed for statistical comparisons.

**Table 2:** Summary of ANOVA for Group, Processing Level, and Session Effects.

Effect	*F*-Value	*df*	*p*-Value	Partial Eta Squared
Group	1.27	2, 33	0.295	0.07
Processing Level	26.21	2, 32	<0.001 *	0.62
Processing Level by Group	2.86	4, 66	0.030 *	0.15

*df* = degrees of freedom. * indicates significance at the 0.05 alpha level.

**Table 3:** Group semantic feature descriptive statistics by processing level.

Processing Level	Group	Mean	SD	Min	Max
Global	ASD	7.50	4.78	0	15
	DLD	8.50	3.94	1	17
	TLD	4.42	2.15	0	8
Local	ASD	3.00	3.25	0	9
	DLD	2.33	3.92	0	14
	TLD	4.17	5.44	0	17
NA	ASD	10.92	8.77	0	28
	DLD	17.08	12.80	0	40
	TLD	10.17	8.16	0	24

SD = standard deviation.

**Table 4:** Mean number of semantic features for each participant for each processing level.

Participant	Global	Local	NA	Participant	Global	Local	NA	Participant	Global	Local	NA
**ASD01**	4.00	0.00	0.00	**DLD01**	4.00	0.00	12.67	**TLD02**	1.67	4.00	7.67
**ASD02**	3.33	1.00	3.67	**DLD04**	1.33	0.00	5.67	**TLD03**	1.33	2.33	4.67
**ASD03**	1.00	3.00	2.67	**DLD05**	2.33	0.00	4.33	**TLD04**	2.67	0.33	0.33
**ASD04**	3.33	2.67	9.33	**DLD06**	2.67	4.67	13.33	**TLD06**	2.00	1.00	2.33
**ASD05**	0.00	0.00	0.00	**DLD07**	2.67	1.00	6.67	**TLD07**	0.00	0.00	0.00
**ASD06**	5.00	1.67	7.33	**DLD09**	11.00	1.00	4.00	**TLD08**	1.67	1.33	5.33
**ASD07**	2.67	1.67	7.00	**DLD14**	3.00	0.67	9.00	**TLD09**	1.33	5.67	4.00
**ASD09**	4.00	1.33	4.33	**DLD17**	5.67	0.67	6.00	**TLD11**	1.33	1.67	8.00
**ASD10**	1.00	0.00	1.33	**DLD18**	3.00	0.67	5.00	**TLD12**	1.00	0.33	2.67
**ASD11**	0.33	0.00	3.00	**DLD19**	2.67	0.00	3.33	**TLD13**	2.33	0.00	2.00
**ASD12**	3.00	0.67	3.00	**DLD20**	2.67	0.00	0.00	**TLD14**	1.67	0.00	3.67
**ASD16**	2.33	0.00	2.00	**DLD21**	0.33	1.33	1.00	**TLD99**	0.67	0.00	0.00

## Supplementary Materials

The following are available online at https://www.mdpi.com/2076-3425/10/4/231/s1, Children’s story script with all of the corresponding visual images.

## References

[1] Bishop D.V.M. (2010). Overlaps between autism and language impairment: Phenomimicry or shared etiology?. Behav. Genet..

[2] Riches N.G., Loucas T., Baird G., Charman T., Simonoff E. (2012). Interpretation of compound nouns by adolescents with specific language impairment and autism spectrum disorders: An investigation of phenotypic overlap. Int. J. Speech Lang. Pathol..

[3] Tager-Flusberg H., Rice M.L., Warren S.F. (2004). Do autism and specific language impairment represent overlapping language disorders. Developmental Language Disorders: From Phenotypes to Etiologies.

[4] Kjelgaard M.M., Tager-Flusberg H. (2001). An investigation of language impairment in autism: Implications for genetic subgroups. Lang. Cogn. Process..

[5] Weismer S.E., Tomblin J.B., Zhang X.Y., Buckwalter P., Chynoweth J.G., Jones M. (2000). Nonword repetition performance in school-age children with and without language impairment. J. Speech Lang. Hear. Res..

[6] Roberts J.A., Rice M.L., Tager-Flusberg H. (2004). Tense marking in children with autism. Appl. Psycholinguist..

[7] Laws G., Bates G., Feuerstein M., Mason-Apps E., White C. (2012). Peer acceptance of children with language and communication impairments in a mainstream primary school: Associations with type of language difficulty, problem behaviours and a change in placement organization. Child Lang. Teach. Ther..

[8] McGregor K.K., Berns A.J., Owen A.J., Michels S.A., Duff D., Bahnsen A.J., Lloyd M. (2012). Associations between syntax and the lexicon among children with or without ASD and language impairment. J. Autism Dev. Disord..

[9] Leyfer O.T., Tager-Flusberg H., Dowd M., Tomblin J.B., Folstein S.E. (2008). Overlap between autism and specific language impairment: Comparison of autism diagnostic interview and autism diagnostic observation schedule scores. Autism Res..

[10] Bishop D.V.M., Norbury C.F. (2002). Exploring the borderlands of autistic disorder and specific language impairment: A study using standardised diagnostic instruments. J. Child Psychol. Psychiatry Allied Discip..

[11] Whitehouse A.J.O., Barry J.G., Bishop D.V.M. (2008). Further defining the language impairment of autism: Is there a specific language impairment subtype?. J. Commun. Disord..

[12] Eigsti I.M., Bennetto L., Dadlani M.B. (2007). Beyond pragmatics: Morphosyntactic development in autism. J. Autism Dev. Disord..

[13] Williams D., Botting N., Boucher J. (2008). Language in autism and specific language impairment: Where are the links?. Psychol. Bull..

[14] Loucas T., Riches N.G., Charman T., Pickles A., Simonoff E., Chandler S., Baird G. (2010). Speech perception and phonological short-term memory capacity in language impairment: Preliminary evidence from adolescents with specific language impairment (SLI) and autism spectrum disorders (ASD). Int. J. Lang. Commun. Disord..

[15] American Psychiatric Association (2013). Diagnostic and Statistical Manual of Mental Disorders: DSM-5.

[16] Leonard L.B. (2014). Children with Specific Language Impairment.

[17] McPhillips M., Finlay J., Bejerot S., Hanley M. (2014). Motor deficits in children with autism spectrum disorder: A cross-syndrome study. Autism Res..

[18] Fitch A., Fein D.A., Eigsti I.M. (2015). Detail and Gestalt focus in individuals with optimal outcomes from autism spectrum disorders. J. Autism Dev. Disord..

[19] Frith U. (1989). Autism: Explaining the Enigma.

[20] Kuschner E.S., Bodner K.E., Minshew N.J. (2009). Local vs. global approaches to reproducing the Rey Osterrieth complex figure by children, adolescents, and adults with high-functioning autism. Autism Res..

[21] Happé F. (1999). Autism: Cognitive deficit or cognitive style?. Trends Cogn. Sci..

[22] Happé F. (2005). The weak central coherence account of autism. Handbook of Autism and Pervasive Developmental Disorders.

[23] Barnes J.L., Baron-Cohen S. (2012). The big picture: Storytelling ability in adults with autism spectrum conditions. J. Autism Dev. Disord..

[24] Diehl J.J., Bennetto L., Young E.C. (2006). Story recall and narrative coherence of high-functioning children with autism spectrum disorders. J. Abnorm. Child Psychol..

[25] Losh M., Capps L. (2003). Narrative ability in high-functioning children with autism or Asperger’s syndrome. J. Autism Dev. Disord..

[26] Potrzeba E.R., Fein D., Naigles L. (2015). Investigating the shape bias in typically developing children and children with autism spectrum disorders. Front. Psychol..

[27] Tek S., Jaffery G., Fein D., Naigles L.R. (2008). Do children with autism spectrum disorders show a shape bias in word learning?. Autism Res..

[28] Tek S., Jaffery G., Swensen L., Fein D., Naigles L.R. (2012). The shape bias is affected by differing similarity among objects. Cogn. Dev..

[29] Hartley C., Allen M.L. (2014). Brief report: Generalisation of word-picture relations in children with autism and typically developing children. J. Autism Dev. Disord..

[30] Akshoomoff N., Stiles J., Wulfeck B. (2006). Perceptual organization and visual immediate memory in children with specific language impairment. J. Int. Neuropsychol. Soc..

[31] Norbury C.F. (2005). Barking up the wrong tree? Lexical ambiguity resolution in children with language impairments and autistic spectrum disorders. J. Exp. Child Psychol..

[32] Riches N.G., Loucas T., Baird G., Charman T., Simonoff E. (2016). Elephants in pyjamas: Testing the weak central coherence account of autism spectrum disorders using a syntactic disambiguation task. J. Autism Dev. Disord..

[33] Collisson B.A., Grela B., Spaulding T., Rueckl J.G., Magnuson J.S. (2015). Individual differences in the shape bias in preschool children with specific language impairment and typical language development: Theoretical and clinical implications. Dev. Sci..

[34] Samuelson L.K., Smith L.B. (1999). Early noun vocabularies: Do ontology, category structure and syntax correspond?. Cognition.

[35] Landau B., Smith L.B., Jones S.S. (1988). The importance of shape in early lexical learning. Cogn. Dev..

[36] Howlin P. (2003). Outcome in high-functioning adults with autism with and without early language delays: Implications for the differentiation between autism and Asperger syndrome. J. Autism Dev. Disord..

[37] Loucas T., Charman T., Pickles A., Simonoff E., Chandler S., Meldrum D., Baird G. (2008). Autistic symptomatology and language ability in autism spectrum disorder and specific language impairment. J. Child Psychol. Psychiatry.

[38] Younger B. (1990). Infant categorization—Memory for category-level and specific item information. J. Exp. Child Psychol..

[39] Klinger L.G., Dawson G. (2001). Prototype formation in autism. Dev. Psychopathol..

[40] Dunn M., Gomes H., Sebastian M.J. (1996). Prototypicality of responses of autistic, language disordered, and normal children in a word fluency task. Child Neuropsychol..

[41] MacKay G., Shaw A. (2004). A comparative study of figurative language in children with autistic spectrum disorders. Child Lang. Teach. Ther..

[42] Condouris K., Meyer E., Tager-Flusberg H. (2003). The relationship between standardized measures of language and measures of spontaneous speech in children with autism. Am. J. Speech Lang. Pathol..

[43] Cree G.S., McNorgan C., McRae K. (2006). Distinctive features hold a privileged status in the computation of word meaning: Implications for theories of semantic memory. J. Exp. Psychol. Learn. Mem. Cogn..

[44] Norbury C.F., Griffiths H., Nation K. (2010). Sound before meaning: Word learning in autistic disorders. Neuropsychologia.

[45] Lord C., Risi S., Pickles A., Rice M., Warren S.F. (2004). Trajectory of language development in autistic spectrum disorders. Developmental Language Disorders: From Phenotypes to Etiologies.

[46] Happé F. (1997). Central coherence and theory of mind in autism: Reading homographs in context. Br. J. Dev. Psychol..

[47] Jolliffe T., Baron-Cohen S. (1999). A test of central coherence theory: Linguistic processing in high-functioning adults with autism or Asperger syndrome: Is local coherence impaired?. Cognition.

[48] Jolliffe T., Baron-Cohen S. (2000). Linguistic processing in high-functioning adults with autism or Asperger’s syndrome. Is global coherence impaired?. Psychol. Med..

[49] Jolliffe T., Baron-Cohen S. (1997). Are people with autism and Asperger syndrome faster than normal on the embedded figures test?. J. Child Psychol. Psychiatry Allied Discip..

[50] Olu-Lafe O., Liederman J., Tager-Flusberg H. (2014). Is the ability to integrate parts into wholes affected in autism spectrum disorder?. J. Autism Dev. Disord..

[51] Robertson C.E., Thomas C., Kravitz D.J., Wallace G.L., Baron-Cohen S., Martin A., Baker C.I. (2014). Global motion perception deficits in autism are reflected as early as primary visual cortex. Brain.

[52] Shah A., Frith U. (1983). An islet of ability on autistic-children—A research note. J. Child Psychol. Psychiatry Allied Discip..

[53] Shah A., Frith U. (1993). Why do autistic individuals show superior performance on the block design task. J. Child Psychol. Psychiatry Allied Discip..

[54] Soriano M.F., Ibanez-Molina A.J., Paredes N., Macizo P. (2018). Autism: Hard to switch from details to the whole. J. Abnorm. Child Psychol..

[55] Mottron L., Dawson M., Soulieres I., Hubert B., Burack J. (2006). Enhanced perceptual functioning in autism: An update, and eight principles of autistic perception. J. Autism Dev. Disord..

[56] Wang L.X., Mottron L., Berthiaume C., Dawson M. (2007). Local bias and local-to-global interference without global deficit: A robust finding in autism under various conditions of attention, exposure time, and visual angle. Cogn. Neuropsychol..

[57] Booth R., Happé F. (2010). “Hunting with a knife and …fork”: Examining central coherence in autism, attention deficit/hyperactivity disorder, and typical development with a linguistic task. J. Exp. Child Psychol..

[58] Fein D., Barton M., Eigsti I.M., Kelley E., Naigles L., Schultz R.T., Stevens M., Helt M., Orinstein A., Rosenthal M. (2013). Optimal outcome in individuals with a history of autism. J. Child Psychol. Psychiatry.

[59] Stevens M.C., Fein D.A., Dunn M., Allen D., Waterhouse L.H., Feinstein C., Rapin I. (2000). Subgroups of children with autism by cluster analysis: A longitudinal examination. J. Am. Acad. Child Adolesc. Psychiatry.

[60] Kourkoulou A., Leekam S.R., Findlay J.M. (2012). Implicit learning of local context in autism spectrum disorder. J. Autism Dev. Disord..

[61] Gladfelter A., Goffman L., Benham S., Steeb A. Extended word learning in children with developmental language disorder.

[62] Gladfelter A., Goffman L. (2018). Semantic richness and word learning in children with autism spectrum disorder. Dev. Sci..

[63] Gladfelter A., Barron K.L., Johnson E. (2019). Visual and verbal semantic productions in children with ASD, DLD, and typical language. J. Commun. Disord..

[64] Buchner A., Erdfelder E., Faul F., Lang A.-G. (2017). G*Power: Statistical Power Analyses for Windows and Mac.

[65] Faul F., Erdfelder E., Lang A.-G., Buchner A. (2007). G*Power: A flexible statistical power analysis program for the social, behavioral, and biomedical sciences. Behav. Res. Methods.

[66] Luyster R.J., Kadlec M.B., Carter A., Tager-Flusberg H. (2008). Language assessment and development in toddlers with autism spectrum disorders. J. Autism Dev. Disord..

[67] Williams K. (2007). Expressive Vocabulary Test-II.

[68] McGregor K.K., Oleson J., Bahnsen A., Duff D. (2013). Children with developmental language impairment have vocabulary deficits characterized by limited breadth and depth. Int. J. Lang. Commun. Disord..

[69] Hudry K., Leadbitter K., Temple K., Slonims V., McConachie H., Aldred C., Howlin P., Charman T., Consortium P. (2010). Preschoolers with autism show greater impairment in receptive compared with expressive language abilities. Int. J. Lang. Commun. Disord..

[70] Dunn L.M., Dunn L.M. (2007). Peabody Picture Vocabulary Test.

[71] Lord C., Rutter M., DiLavore P.C., Risi S., Gotham K., Bishop S.L. (2012). ADOS-2 Autism Diagnostic Observation Schedule.

[72] Dawson J., Stout C., Eyer J. (2003). Structured Photographic Expressive Language Test.

[73] Semel E., Wiig E.H., Secord W.A. (2003). Clinical Evaluation of Language Fundamentals.

[74] Benham S., Goffman L., Schweickert R. (2018). An application of network science to phonological sequence learning in children with developmental language disorder. J. Speech Lang. Hear. Res..

[75] Saletta M., Goffman L., Ward C., Oleson J. (2018). Influence of language load on speech motor skill in children with specific language impairment. J. Speech Lang. Hear. Res..

[76] Vuolo J., Goffman L., Zelaznik H.N. (2017). Deficits in coordinative bimanual timing precision in children with specific language impairment. J. Speech Lang. Hear. Res..

[77] Vuolo J., Goffman L. (2018). Language skill mediates the relationship between language load and articulatory variability in children with language and speech sound disorders. J. Speech Lang. Hear. Res..

[78] Dawson J., Stout C., Eyer J., Tattersall P., Fonkalsrud J., Croley K. (2007). Structured Photographic Expressive Language Test—Preschool.

[79] Plante E., Vance R. (1994). Selection of preschool language tests: A data-based approach. Lang. Speech Hear. Serv. Sch..

[80] Greenslade K.J., Plante E., Vance R. (2009). The diagnostic accuracy and construct validity of the structured photographic expressive language test-preschool: Second edition. Lang. Speech Hear. Serv. Sch..

[81] Schopler E., Van Bourgondien M., Wellman G.J., Love S.R. (2010). Childhood Autism Rating Scale.

[82] Hollich G., Jusczyk P.W., Luce P.A. Lexical neighborhood effects in 17-month-old word learning. Proceedings of the 26th Annual Boston-University Conference on Language Development, Boston University.

[83] Storkel H.L. (2001). Learning new words: Phonotactic probability in language development. J. Speech Lang. Hear. Res..

[84] Boersma P., Weenink D. (2012). Praat: Doing Phonetics by Computer.

[85] McGregor K.K., Sheng L., Ball T. (2007). Complexities of expressive word learning over time. Lang. Speech Hear. Serv. Sch..

[86] Navon D. (1977). Forest before trees—Precedence of global Features in visual-perception. Cogn. Psychol..

[87] Guy J., Mottron L., Berthiaume C., Bertone A. (2019). A developmental perspective of global and local visual perception in autism spectrum disorder. J. Autism Dev. Disord..

[88] Koldewyn K., Jiang Y.V., Weigelt S., Kanwisher N. (2013). Global/Local processing in autism: Not a disability, but a disinclination. J. Autism Dev. Disord..

[89] Schlosser R.W. (2007). Appraising the quality of systematic reviews. Focus Tech. Briefs.

[90] Hallgren K.A. (2012). Computing inter-rater reliability for observational data: An overview and tutorial. Tutor. Quant. Methods Psychol..

[91] Skwarchuk S.-L., Anglin J.M. (1997). Expression of superordinates in children’s word definitions. J. Educ. Psychol..

[92] Gastgeb H.Z., Strauss M.S., Minshew N.J. (2006). Do individuals with autism process categories differently? The effect of typicality and development. Child Dev..

[93] Marinellie S.A., Johnson C.J. (2002). Definitional skill in school-age children with specific language impairment. J. Commun. Disord..

[94] McGregor K.K., Appel A. (2002). On the relation between mental representation and naming in a child with specific language impairment. Clin. Linguist. Phon..

[95] McGregor K.K., Bean A. (2012). How children with autism extend new words. J. Speech Lang. Hear. Res..

[96] Tager-Flusberg H. (1985). Basic level and superordinate level categorization by autistic, mentally retarded, and normal children. J. Exp. Child Psychol..

[97] Hansen M.B., Markman E.A. (2009). Children’s use of mutual exclusivity to learn labels for parts of objects. Dev. Psychol..

[98] Kobayashi H. (1998). How 2-year-old children learn novel part names of unfamiliar objects. Cognition.

[99] Markman E.M. (1994). Constraints on word meaning in early language-acquisition. Lingua.

[100] Hall J., McGregor K.K., Oleson J. (2017). Weaknesses in lexical-semantic knowledge among college students with specific learning disabilities: Evidence from a semantic fluency task. J. Speech Lang. Hear. Res..

