# Supplementary material for: How Children with Autism Spectrum Disorder, Developmental Language Disorder, and Typical Language Learn to Produce Global and Local Semantic Features
Source: Brain Sci. 2020 Apr 11;10(4):231. doi: 10.3390/brainsci10040231 (PMC7226439; doi:10.3390/brainsci10040231)
Supplement: Supplementary file 1 [file brainsci-10-00231-s001.pdf]

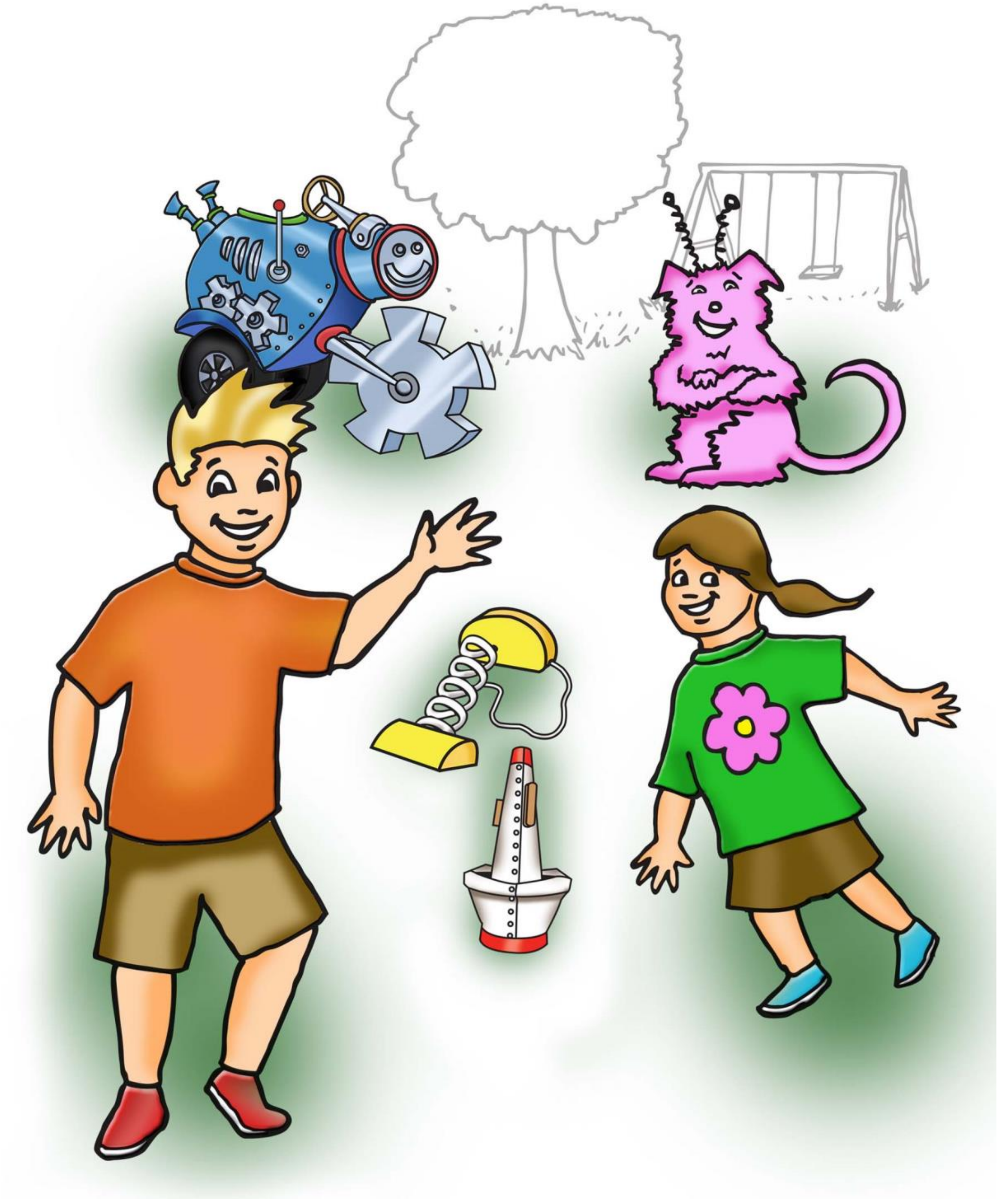

Picture 1. (Picture of Big Brother and Little Sister playing in backyard. In backyard are both stimuli and 2 foils) Narrator: Big Brother and Little Sister like to play outside together.

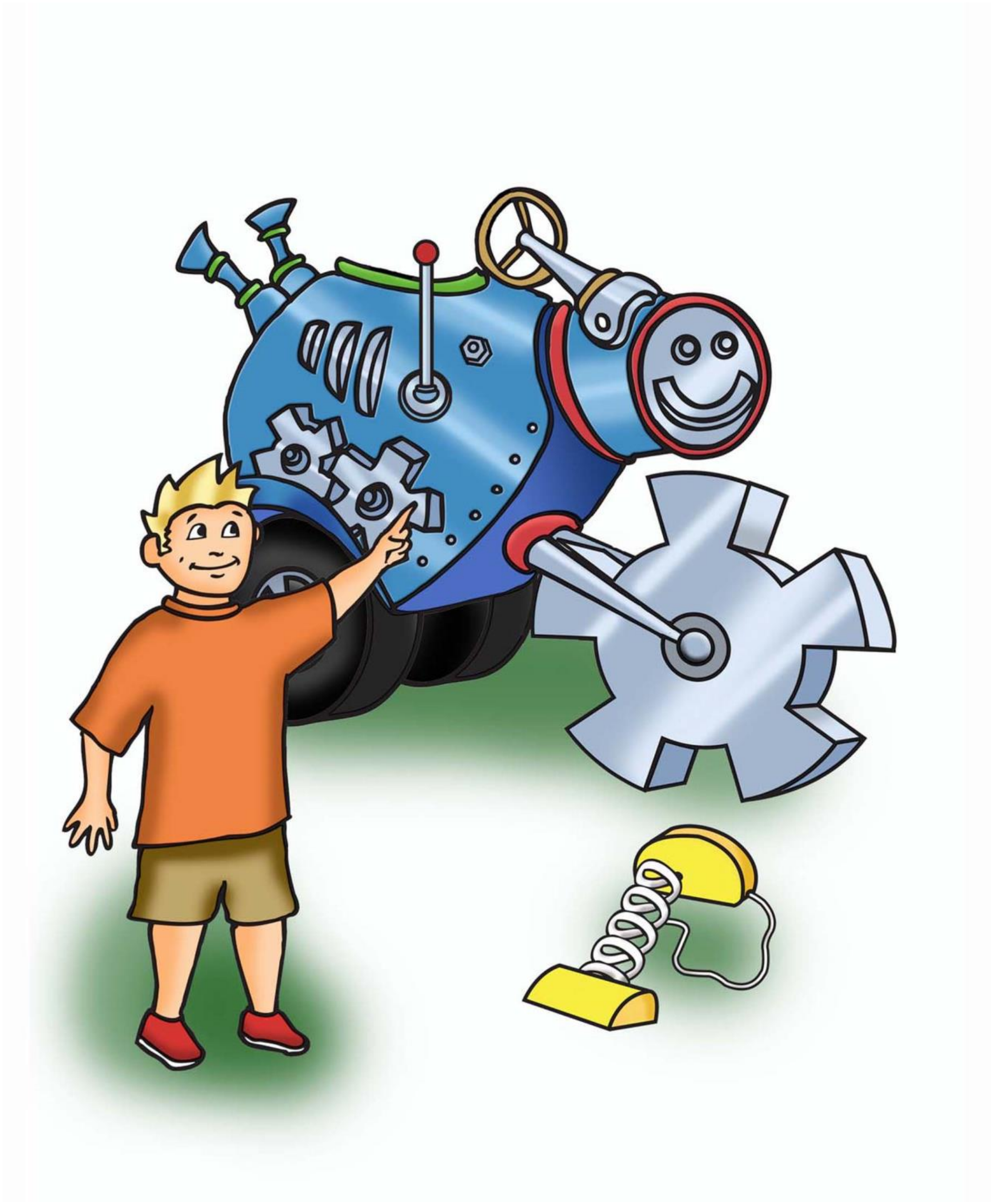

<u>Picture 2.</u> (Big Brother with item 1 and a foil item in background). Narrator: Big Brother said, "I like my _________. It's my favorite!"

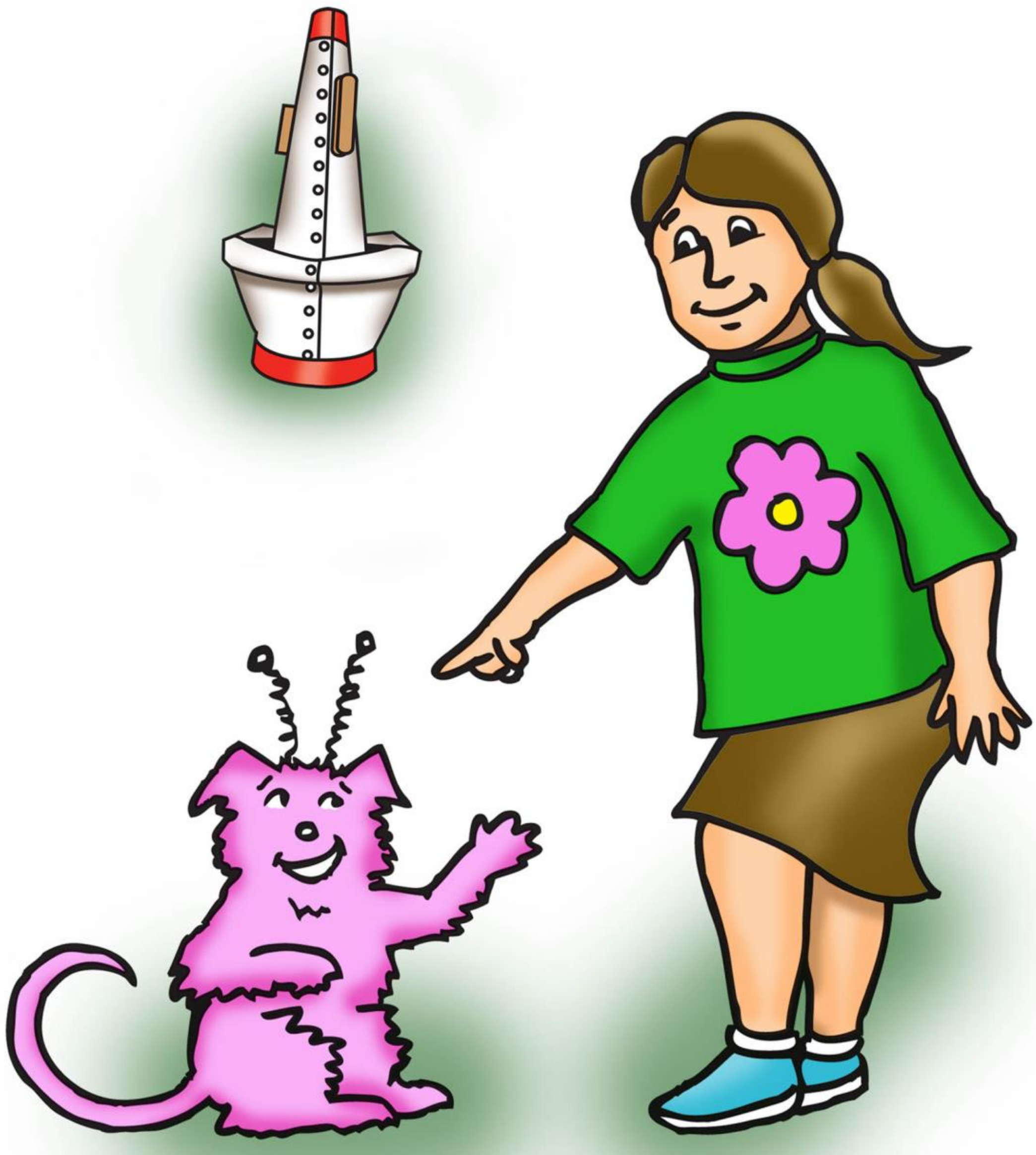

<u>Picture 3.</u> (Little Sister with item 2 and a foil item in background). Narrator: Little Sister said, "I like my __________. It's my favorite!"

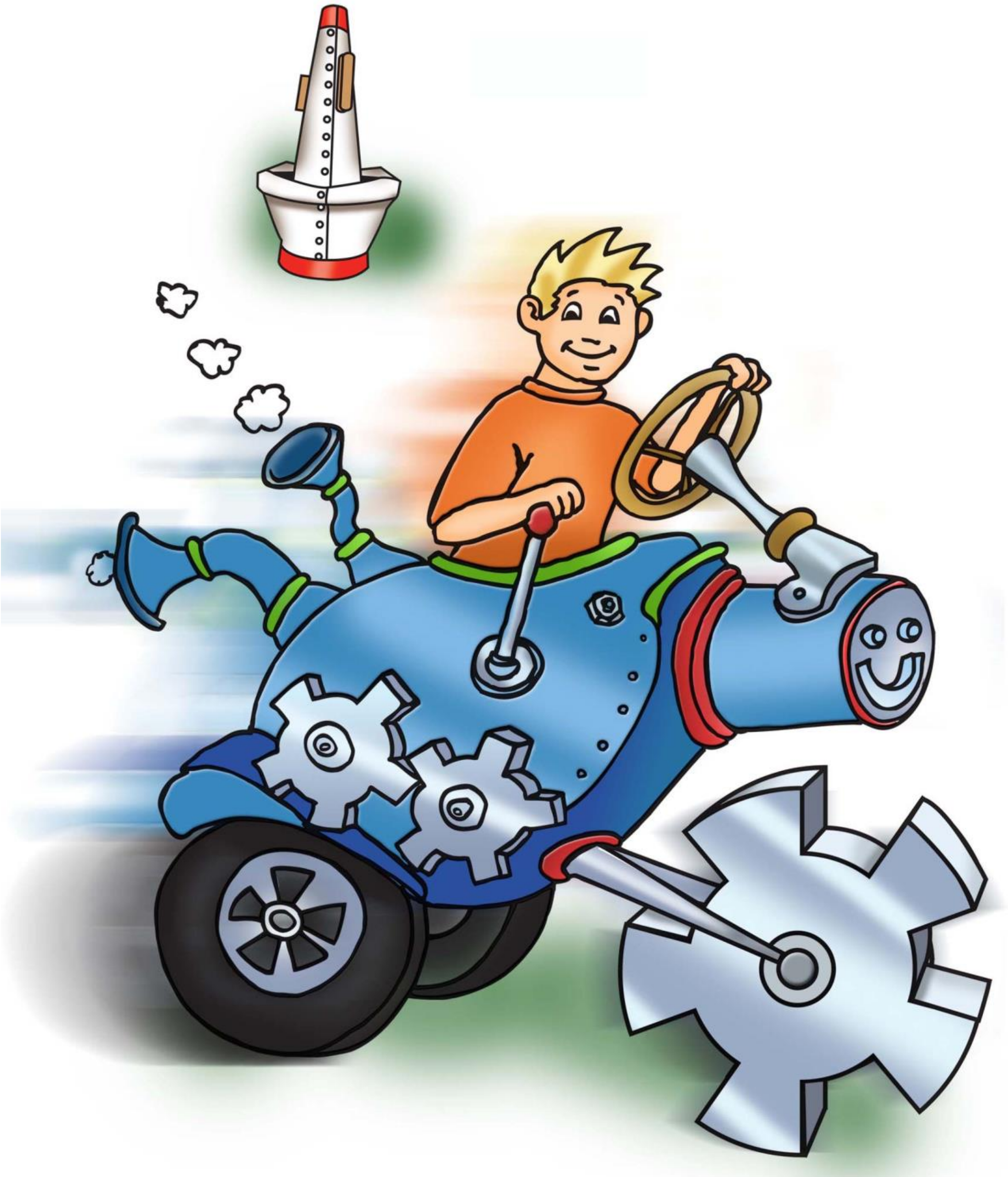

<u>Picture 4.</u> (Big Brother riding item 1 and foil in background). Narrator: Big Brother said, “My

_______ is blue and big. I ride my _____. My ________ drives fast!”

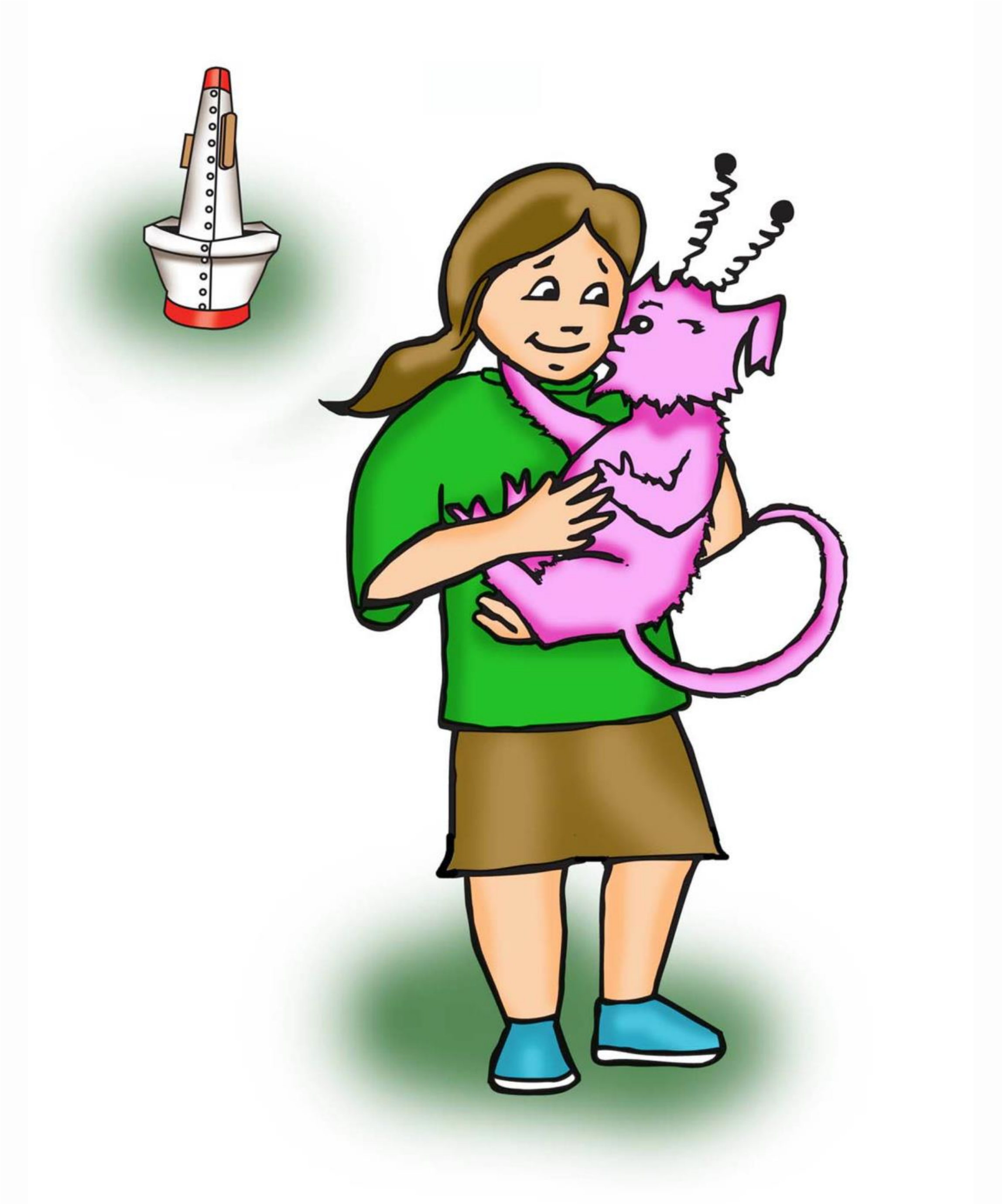

<u>Picture 5</u>. (Little Sister petting item 2 and item 2 is kissing her. Foil in background). Narrator: Little Sister said, “My ______ is soft and small. I cuddle my _______. My ________ gives kisses!

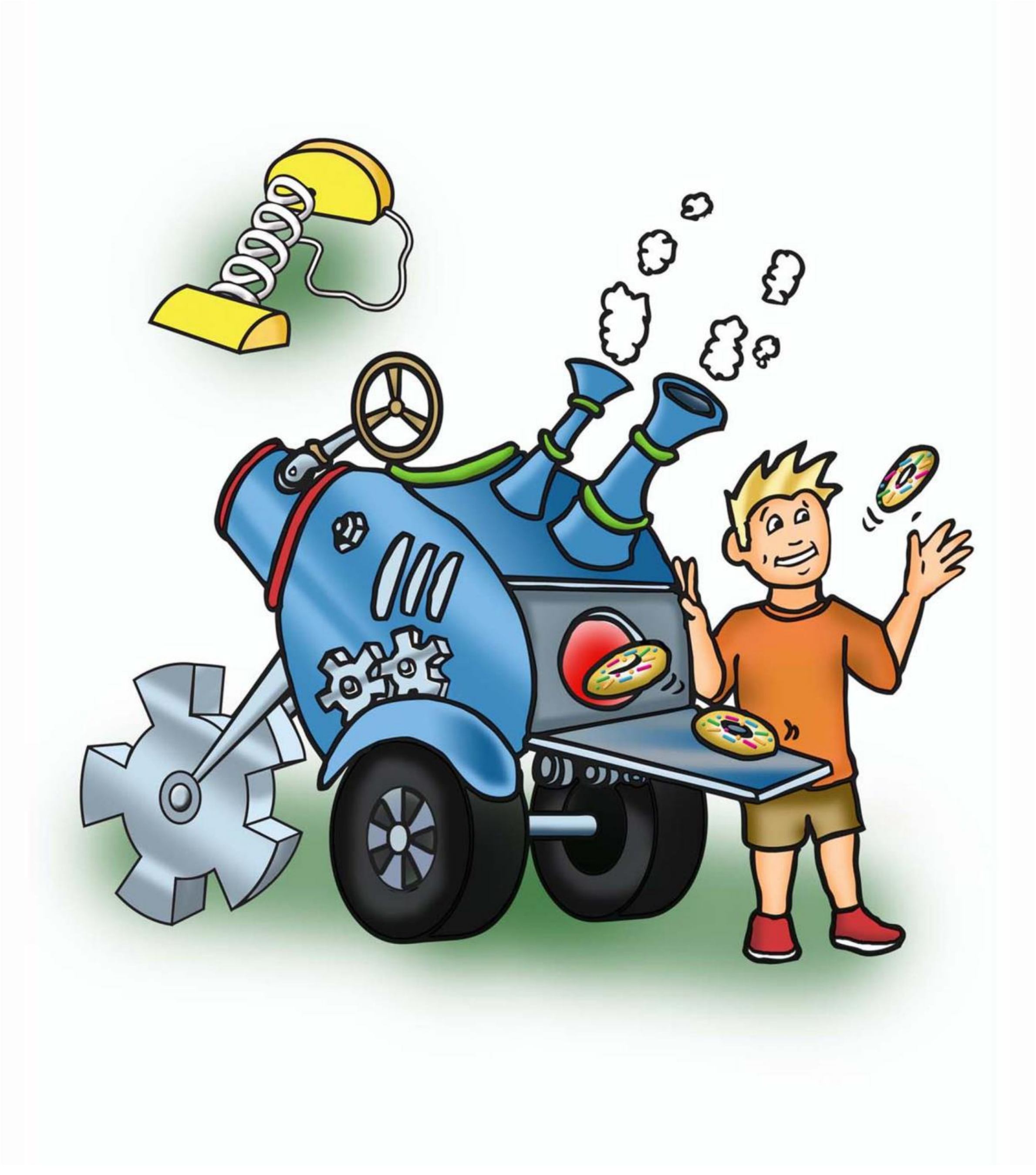

<u>Picture 6.</u> (Big Brother standing next to item 1 and catching donuts shooting out the back of item 1 and foil in background). Narrator: Big Brother said, “My _______ makes donuts! Watch me catch them!”

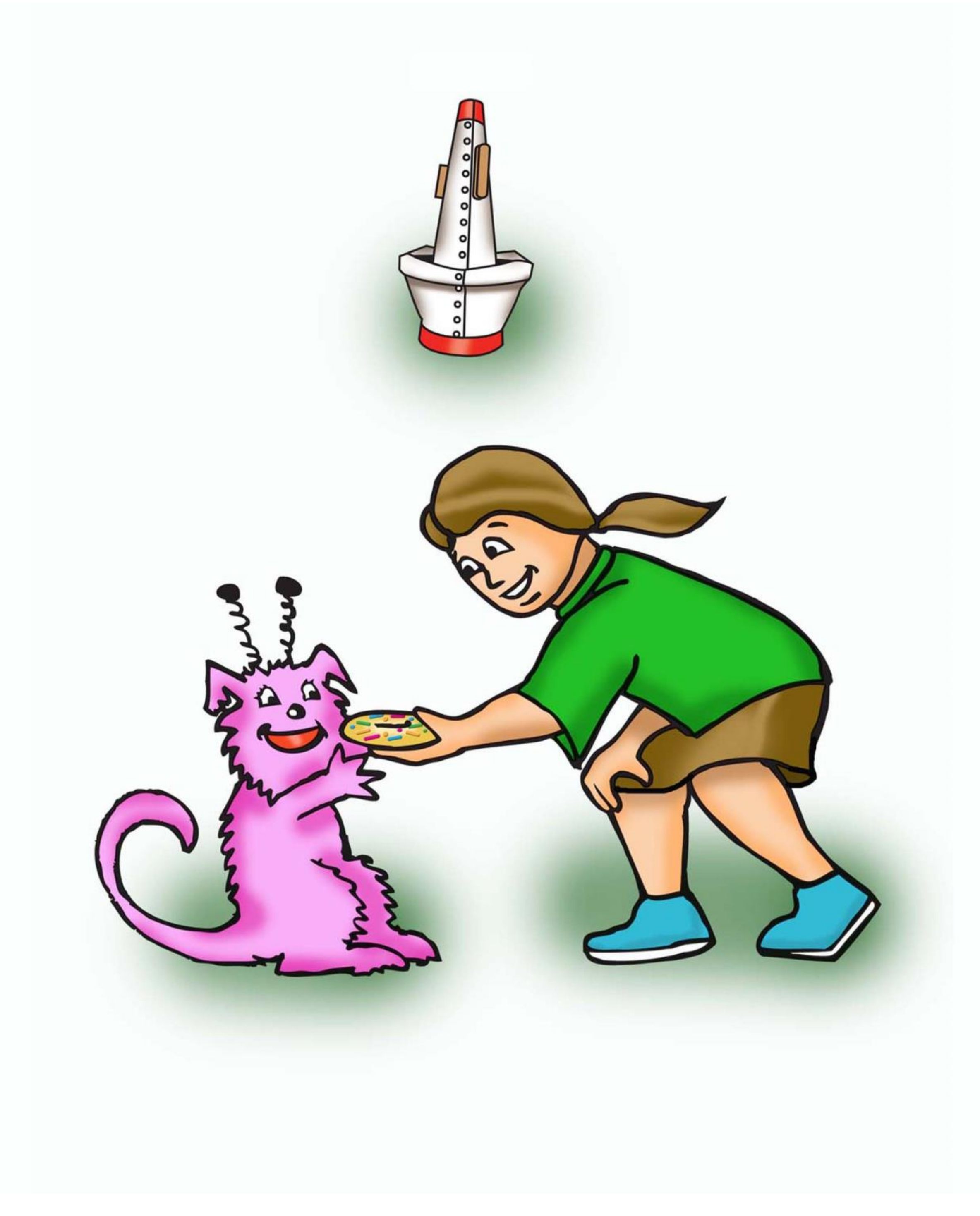

Picture 7. (Little Sister feeding a donut to item 2 and foil in background). Narrator: Little Sister said, “My _________ eats donuts! Watch me feed it!”

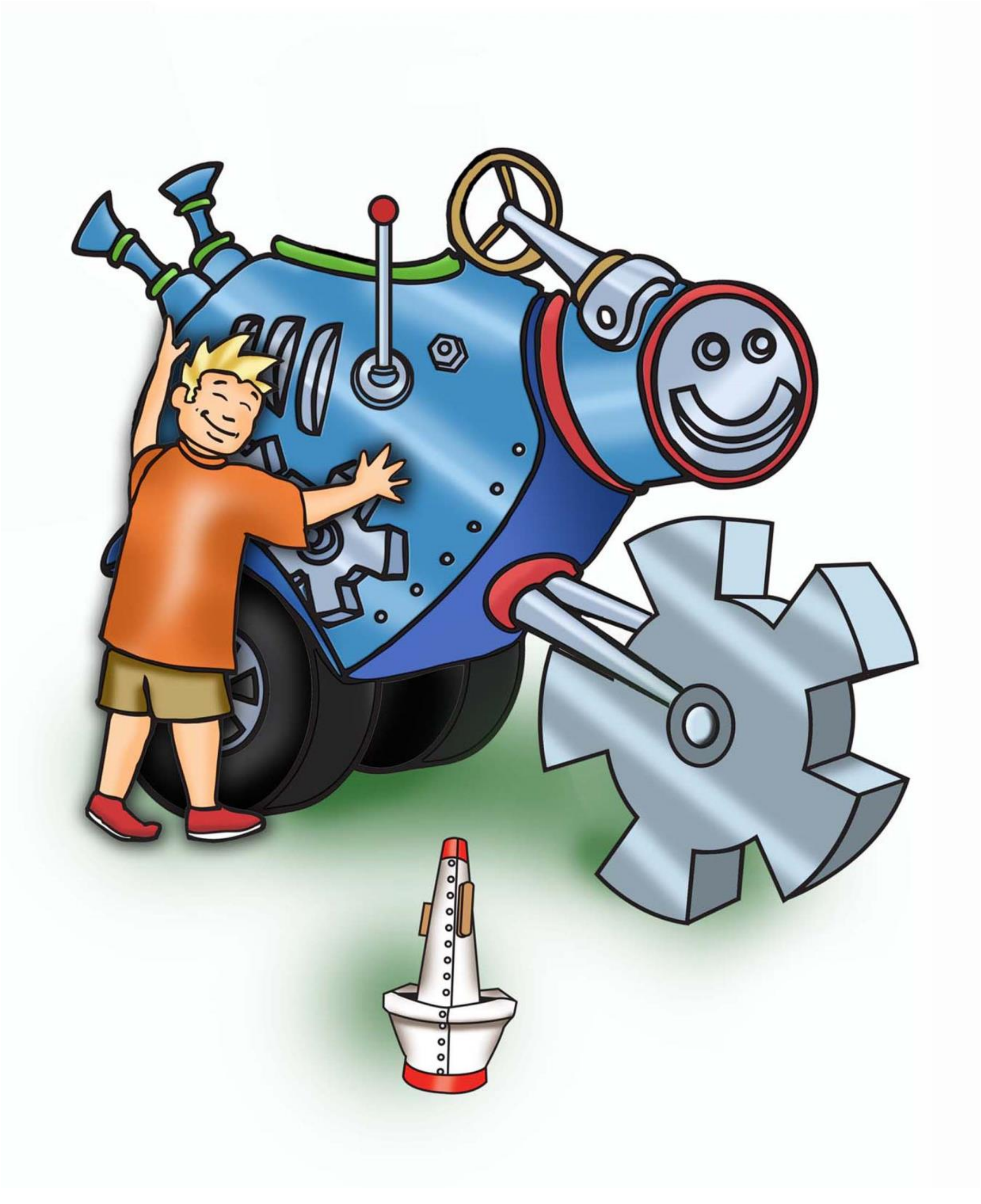

<u>Picture 8.</u> (Big Brother hugging item 1 with foil item in background.) Narrator: "My _____ is shiny. I like my _________," said Big Brother.

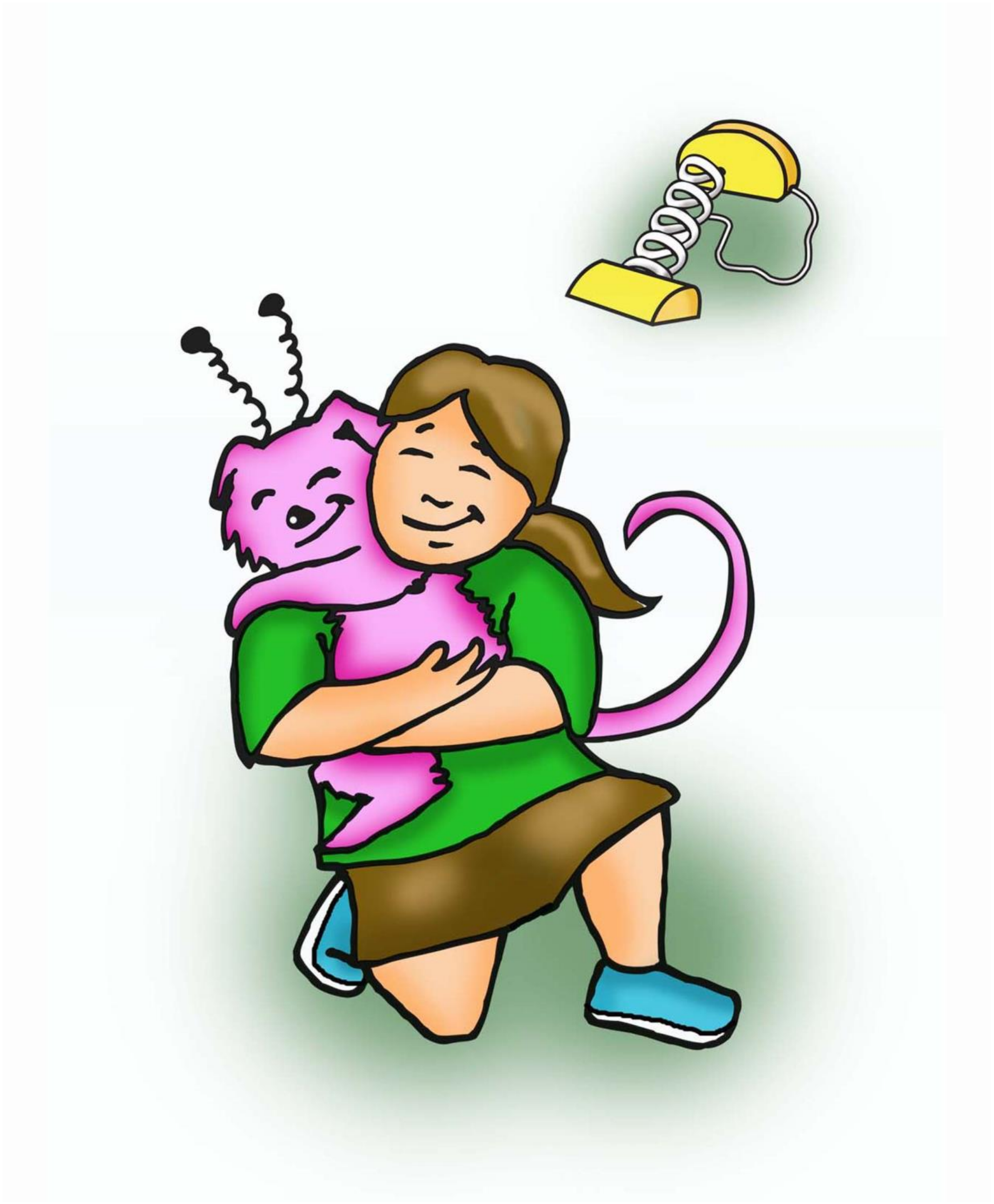

Picture 9. (Little Sister hugging item 2 with foil in background). Narrator: “My _____ is furry. I like my _________,” said Little Sister.

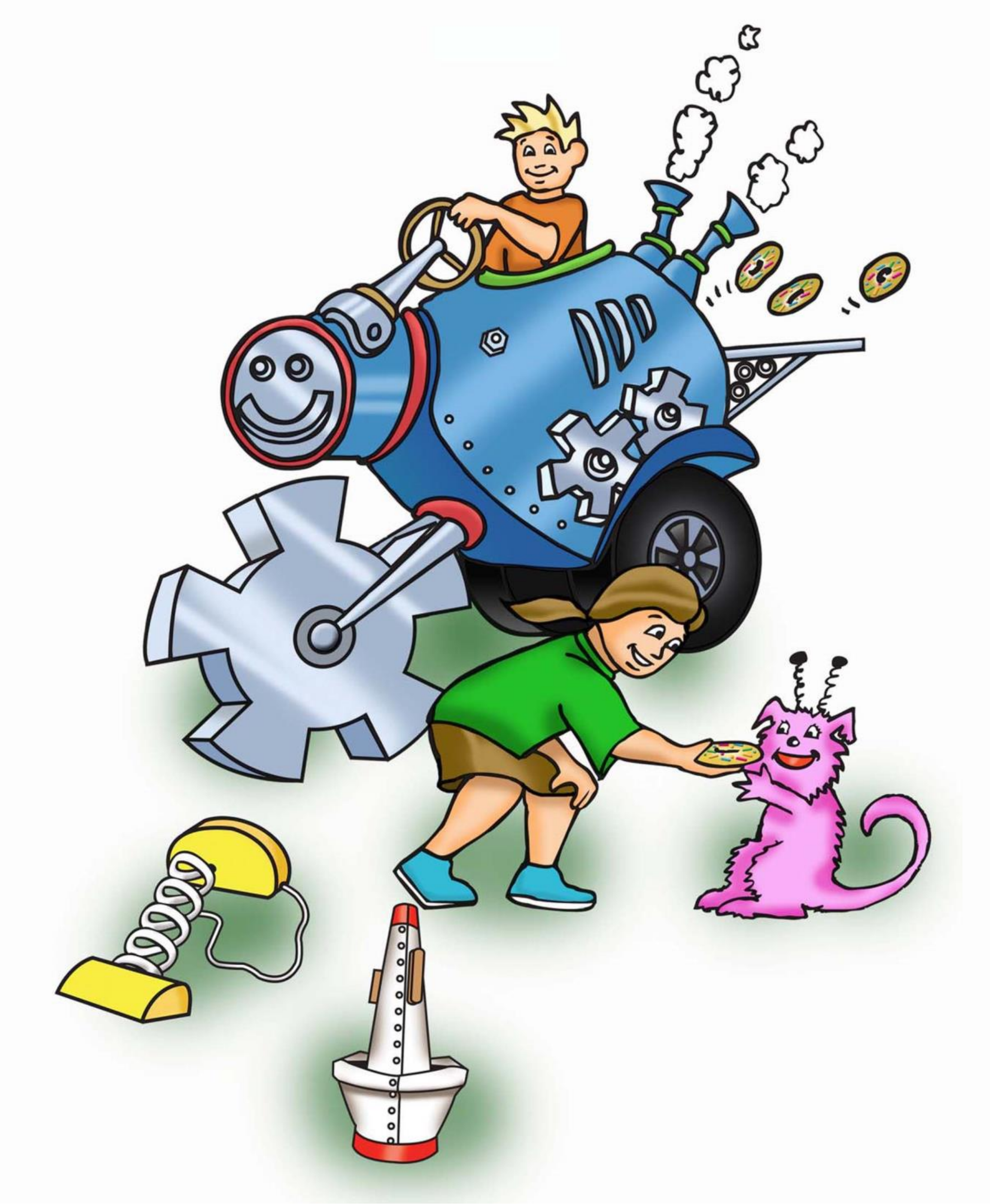

Picture 10. (Big Brother and Little Sister playing in backyard. Item 1 is shooting donuts with Big Brother riding it and Little Sister is feeding item 2 a donut) Narrator: Big Brother and Little Sister like to play outside together.
